# Coeliac disease: to classify or not to classify – that is the question! 

**Published:** 2016

**Authors:** Arzu Ensari

**Affiliations:** *Department of Pathology, Ankara University Medical School, Sihhiye 06100, Ankara, Turkey*

I have followed the discussion on the classification of coeliac disease (CD) published in the autumn issue of Gastroenterology and Hepatology from Bed to Bench with great interest. In my opinion, the ongoing debate on CD focuses around three issues: firstly, do we need a duodenal biopsy for the diagnosis of CD? Secondly, is it necessary to classify the mucosal pathology? Thirdly, which classification is the most reproducible and useful one for clinical assessment of the patient? Regarding the abovementioned issues, below, is the summary of my view, as a pathologist. We have reached the era where the duodenal biopsy is no longer the gold standard for the diagnosis of CD. We need new tools like mucosal tTG antibodies to diagnose cases sensitized to gluten with normal intestinal mucosa or “microscopic enteritis”. However, duodenal biopsy is still considered essential in the diagnostic work-up of adult CD and cannot totally be replaced by serology in many clinical settings. 

Since there is no correlation between degree of mucosal damage and severity of clinical symptoms in CD, classification of mucosal pathology may seem totally irrelevant from a clinical point of view. However, from a pathologist’s point of view, intraepithelial lymphocytosis and flat mucosa are distinct forms (“grades”) of mucosal pathology and may be caused by a variety of entities besides CD. Therefore, the type and degree of mucosal pathology should be reported in a descriptive manner. If pathologists wish to classify the mucosal pathology, a standardized, reproducible, logical and preferably simple classification scheme is essential to avoid unnecessary confusion. The clinician should know the histopathologic appearance of the mucosa, to relate serology and other laboratory findings to the clinical picture and make a differential diagnosis, . 

**Figure 1:**
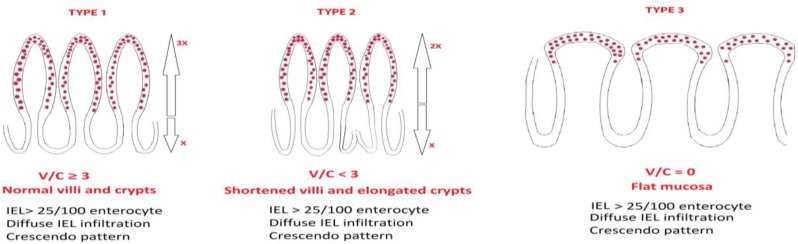
Ensari classification of mucosal pathology in coeliac disease

Marsh, who is a gastroenterologist, has pioneered classifying CD after years of experimental and clinical research and defined the spectrum of mucosal pathology in CD (1). I believe, even Marsh did not expect his scheme to receive such a warm welcome from practising pathologists and become very popular in the evaluation of intestinal biopsies. However, it is interesting that subsequent modifications of the original Marsh classification were proposed by pathologists including Oberhuber (2), Corazza Villanacci (3), and Ensari (4). Unfortunately, Oberhuber’s modification (2) did not gain much popularity among pathologists since it was based on subjective definitions such as “minor or moderate degrees of shortening and blunting of the villi” or “short tent-like remainders of the villi”. This prompted a new classification by Corazza and Villanaci (3), who classified coeliac lesions as grade A (non-atrophic), and grade B (atrophic) in a more simplified yet subjective manner. Difficulty in the interpretation of mucosal pathology in CD has encouraged me to come up with a simplified version of the original Marsh that employed objective morphometric parameters like v/c ratio, as well as IEL count and distribution (4). The “new” version of the “old” classification had two main arguments: firstly, following the steps of Marsh it avoided the term “atrophy” to define villous shortening. Dynamic studies have shown that the mucosa demonstrates a hyperplastic state characterized by elongation of the crypts and widening of the lamina propria by inflammation, both of which reflect the underlying pathophysiology (5). Secondly, Marsh type 2 lesion was redefined. In its original form, Marsh type 2 lesion is almost never seen in routine biopsies unlike Marsh’s dynamic gluten challenge studies. In a real life, however, when there is crypt hyperplasia, villi look shortened due to the decreased v/c ratio compared to mucosae without crypt hyperplasia. These arguments formed the basis of the new version of Marsh classification in which types 1 and 3 were identical to original Marsh, namely intraepithelial lymphocytosis and flat mucosa, respectively. However, type 2 was redefined as mucosa with (any degree of) villous shortening and crypt hyperplasia as well as intraepithelial lymphocytosis (4). The aim was to have a simpler and practical scheme based on objective morphometric parameters that would improve diagnostic accuracy of the pathologists in all settings, both community practice and academic environment. SEM observations in Marsh’s recent paper (6) argue against the presence of intermediate stages of villous shortening/flattening. However, light microscopic images of vertically oriented biopsies very clearly demonstrate that the reverse is true. There are cases with completely flat mucosa and no visible villi, and cases with visible but shortened villi. These intermediary changes of the villi are frequently observed in treated coeliac patients, particularly if they are biopsied sooner during gluten-free diet. More appropriately, type 2 lesion could be defined as “poorly formed” rather than “shortened” villi since the underlying mechanism is likely to involve intestinal stem cells responsible for the maturation and differentiation of crypt epithelial cells in their upward migration to form the intestinal villi. I believe our interpretation of coeliac pathology will improve as stem cell research takes part in the game in the near future. Today, however, my clinical colleagues, both adult and paediatric gastroenterologists will continue to take duodenal biopsies and I shall continue to classify mucosal pathology using my version of Marsh classification!
